# Giant Darier–Ferrand dermatofibrosarcoma protuberans of the abdomen and pelvis: a case report

**DOI:** 10.1186/s13256-021-02687-8

**Published:** 2021-03-15

**Authors:** A. Triki, M. Aloui, M. Ghalleb, I. Jbir, Ben Naceur, J. Ben Hassouna, R. Chargui, K. Rahal

**Affiliations:** 1 Surgical Oncology Department, Salah Azaiez Institute of Oncology, Tunis, Tunisia; 2 Plastic Surgery Unit, Salah Azaiez Institute of Oncology, Tunis, Tunisia; 3 Faculty of Medicine, Tunis, Tunisia

**Keywords:** Darier–Ferrand dermatofibrosarcoma, Recurrent, Surgical resection

## Abstract

**Background:**

Darier–Ferrand dermatofibrosarcoma (DFS) is a rare malignant cutaneous neoplasm characterized by local aggressiveness, high risk of recurrence, and low metastatic potential.

**Case presentation:**

A 60-year-old Tunisian man with recurrent abdominopelvic DFS for which he had undergone multiple excisions presented with an extensive DFS that invaded the external genitals. He underwent a large excision with emasculation and thin cutaneous graft of the abdominal wall and local skin flap in the pelvis.

**Conclusion:**

DFS is a rare yet recurrent skin tumor. Wide excision with free margins remains the cornerstone of treatment. We report a case of a giant DFS treated with wide excision and reconstructive surgery to cover the defect.

## Background

Darier–Ferrand dermatofibrosarcoma (DFS) is a rare skin tumor, representing only 0.1% of malignant cutaneous neoplasms. DFS is usually characterized by high recurrence, slow growth, and low metastatic potential [1]. It occurs predominantly in adults at any age, but especially between their second and fifth decades of life. The trunk wall and proximal extremities are the most common locations in DFS (50%) [2]. Complete surgical resection is considered the gold-standard therapy. The potential for recurrence of DFS is directly related to the margin of resection.

We report herein a case of a giant and highly advanced DFS and shed additional light on a rare but dreadful disease.

## Case presentation

We present the case of a 60-year-old Tunisian man with a medical history of recurrent abdominopelvic DFS for 35 years, for which he had undergone ten surgical excisions in another clinic. Not all the histologic reports were available, but for those that were found, the margins were free of tumor. He presented with a giant DFS of the abdominal wall and pelvis. The biopsy confirmed the diagnosis and the patient was referred to our outpatient clinic.

**Fig. 1:**
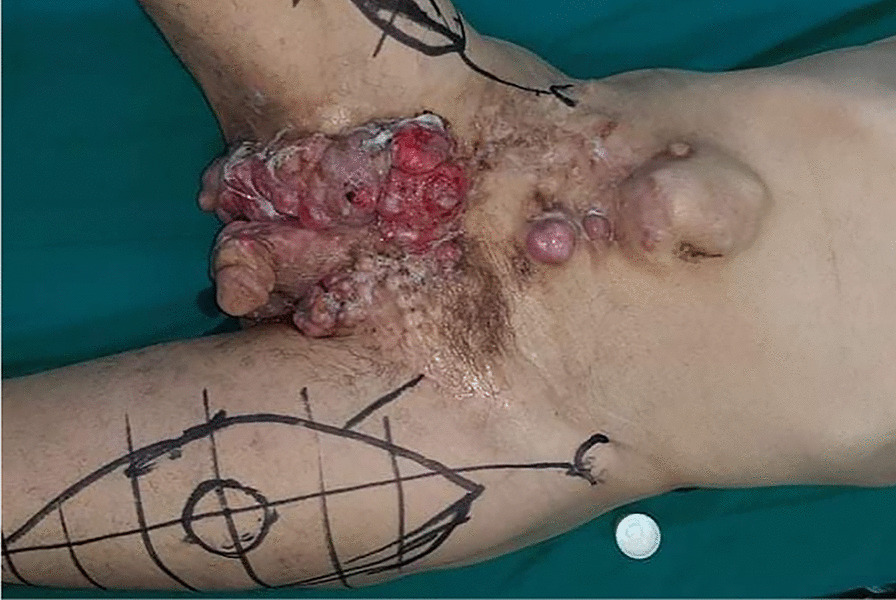
Multiple dermatofibrosarcoma masses extending from the abdominal wall to the pelvis

**Fig. 2:**
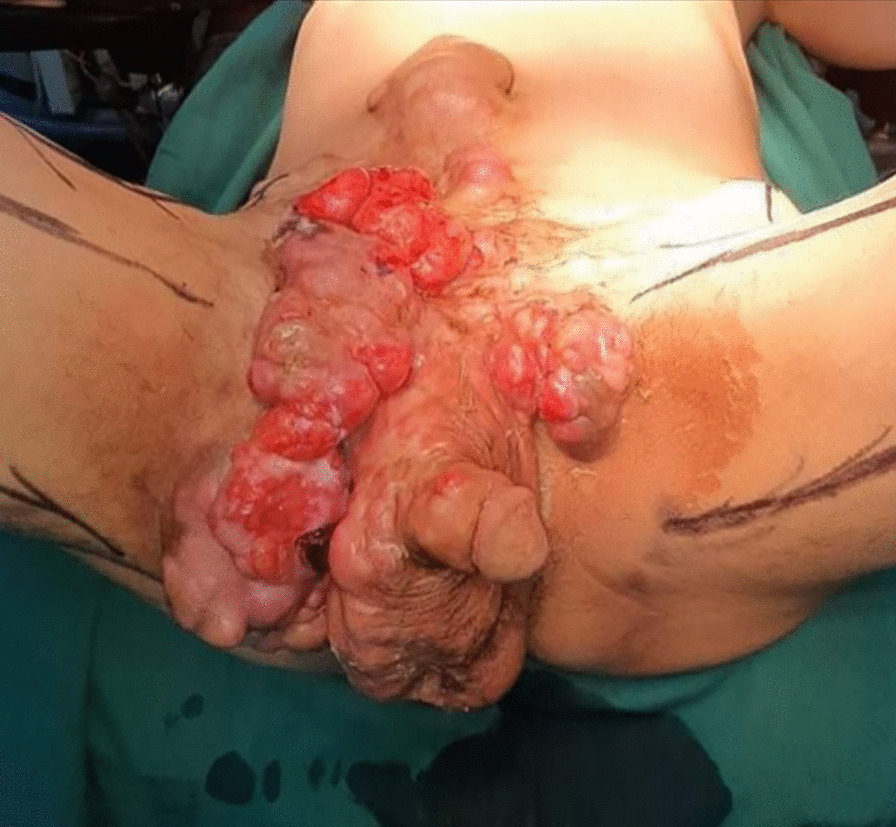
Extended dermatofibrosarcoma of the abdomen and pelvis

**Fig. 3:**
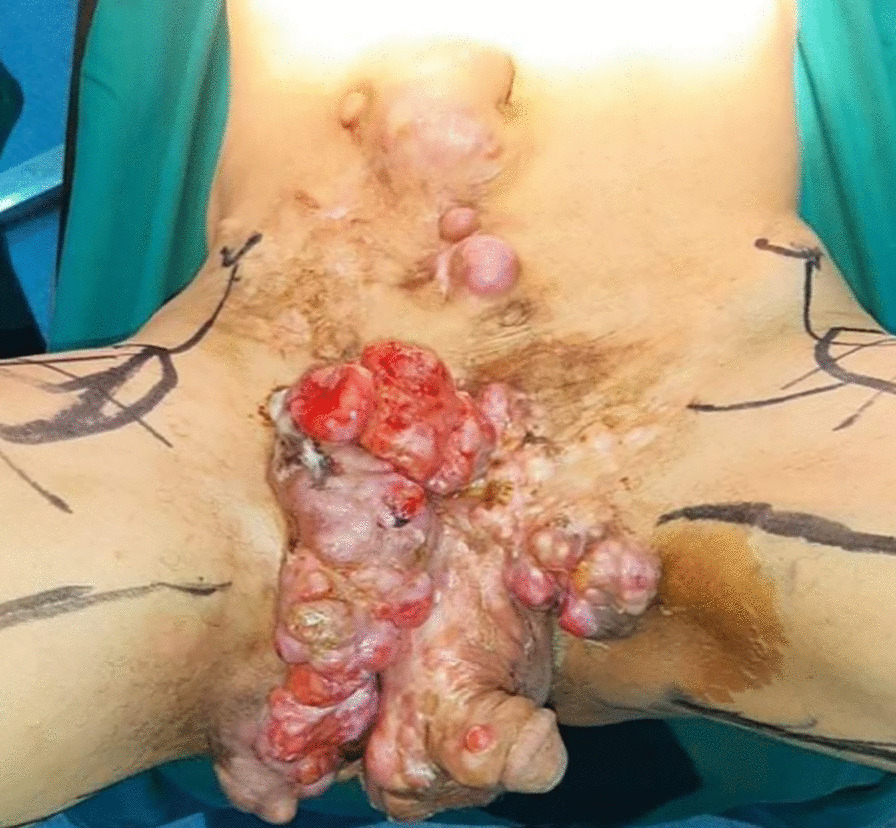
Dermatofibrosarcoma of the abdomen and pelvis invading the right scrotum

The patient had a poor general state; he was afebrile and hemodynamically stable. On physical examination, multiple tumors were observed extending from the abdominal wall to the pelvis and external genitals a distance of 1 cm from the anal margin (Figs. 1, 2, 3).

**Fig. 4:**
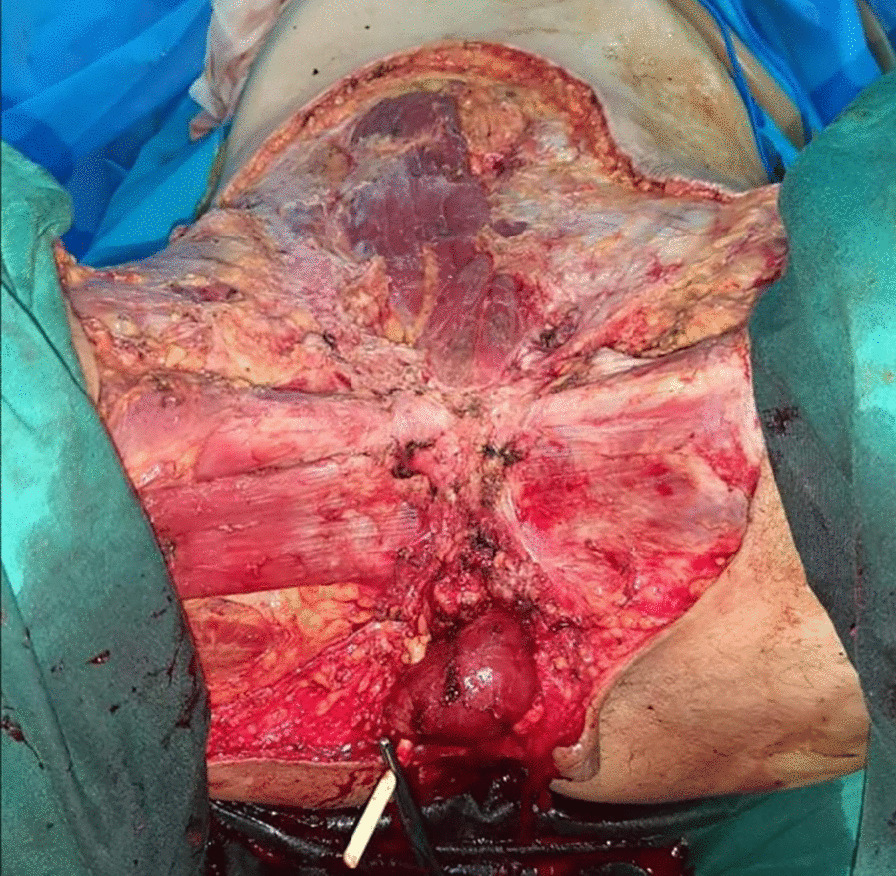
Wide local excision of the dermatofibrosarcoma

**Fig. 5:**
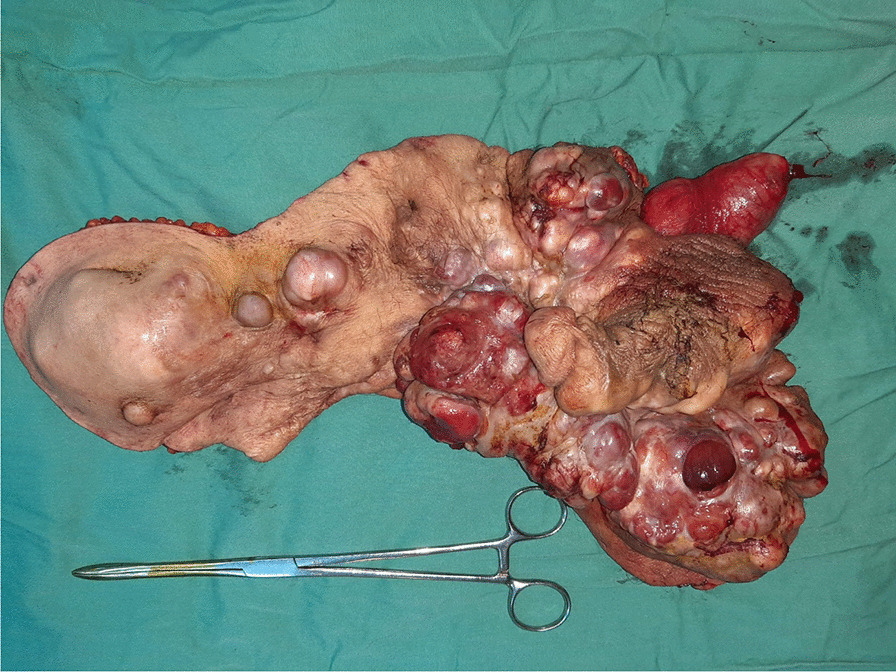
Excised specimen of dermatofibrosarcoma

**Fig. 6:**
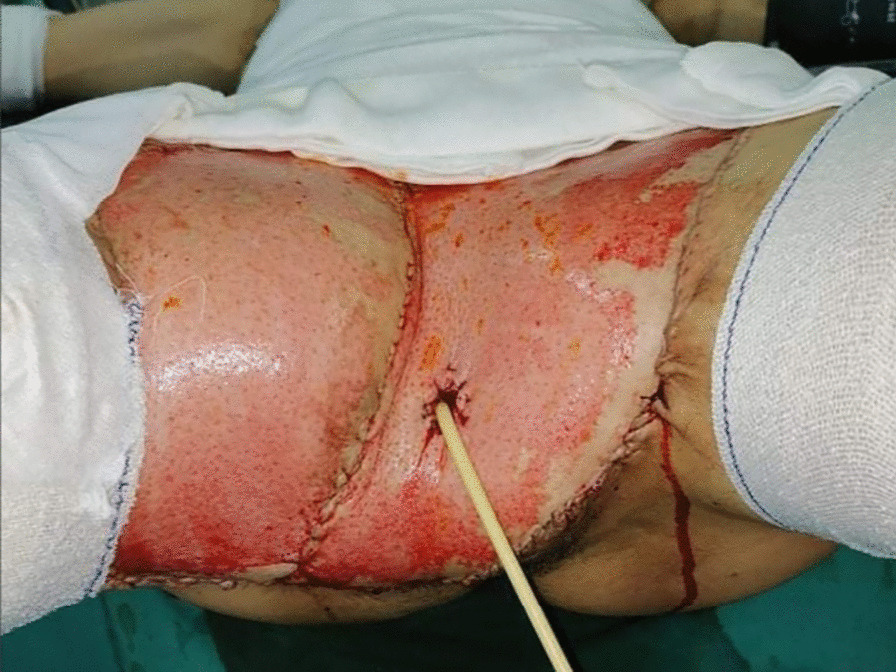
Pelvis reconstruction with local skin flap

A computed tomography scan of the abdomen and pelvis revealed multiple subcutaneous and infiltrative tumor masses of the anterior abdominal wall and pelvis that invaded the right scrotum. No metastases were found. After discussion in a multidisciplinary meeting, we opted for wide local excision combined with emasculation and reconstruction with a local skin flap for the pelvis and thin cutaneous graft for the abdominal wall (Figs. 4, 5, 6).

The surgery lasted 240 minutes. No preoperative complications or blood loss occurred. The excised specimen measured approximately 33 cm × 17 cm × 7 cm in the greatest dimensions. The postoperative course was uneventful. The patient was discharged on day 7. The final histology report showed clear margins.

The case was discussed again in a multidisciplinary meeting and the decision was made for close follow-up every 3 months for the first 2 years. The patient progressed well during 7 months of follow-up. No urinary problems were reported. The only concern was an episode of depression, which was followed up by a psychiatrist.

## Discussion

DFS is a rare mesenchymal tumor, representing 1.8% of all soft tissue sarcomas and only 0.1% of all cancers. It was first described by Taylor in 1890 [3] and then by Darier and Ferrand in 1924. It is characterized by slow infiltrative growth, local aggressiveness, and a high potential for local recurrence if not properly treated. However, distant metastases are extremely rare (≤ 5%) and generally occur as a late sequel after local recurrences [4].

DFS can appear at any age, but most commonly in patients between the ages of 20 and 50 years [3]. Although it appears mostly in adults, various case series report an incidence of 6–20% in children, and congenital cases have even been observed [5]. The most common anatomical site affected by DFS is the trunk (42–72%), with the majority of cases found on the chest and abdomen; 16–30% of DFS cases are located on the proximal extremities (particularly on the legs), and around 16% affect the head and neck area [6, 7].

DFS usually appears as a pink, reddish-brown, or violaceous plaque limited to the skin. With time, the tumor can evolve into multiple "protuberant" nodules that may infiltrate the subcutaneous tissue, fascia, muscles, and even bone [8]. In our case, no infiltration of the adjacent muscular structures was evident, but the tumor had invaded the right scrotum. On ultrasound, DFS is mostly hypoechoic or mixed hyperechoic, with well-defined or irregular margins. Vascularity of DFS, which is a marker of malignancy, varies as well [9]. This happened in our case, where a preoperative ultrasound found multiple hypoechoic and vascularized nodules of the right testicle.

DFS is characterized by diffuse infiltration of the dermis with a low rate of mitosis and minimal atypia. Aggressive clinical behavior is correlated with the mitotic count, necrosis, and areas of fibrosarcomatous change. Tumor cells demonstrate strong positive staining for vimentin, CD34, apolipoprotein D, and nestin when subjected to immunohistochemical studies. Negative staining is found for desmin, S100 protein, factor XIIIa (FXIIIa), stromelysin-3, HMGA1 and HMGA2, tenascin, podoplanin (D2-40), CD163, and keratins [10].

The treatment of choice is wide local excision, with negative margins of 3–5 cm from the tumor edge including the skin, the subcutaneous tissue, and the underlying fascia [11].

Mohs micrographic surgery is an alternative to wide excision that is considered by some as the preferred treatment for DFS. It consists of a removal method that offers precise microscopic control of the entire tumor margin while maximizing the conservation of healthy tissue [12, 13]. It is performed by removing a thin margin of tissue circumferentially around and deep to the clinical tumor that is then examined under a microscope. The process is repeated until the tumor has negative histologic margins.

Our patient underwent wide excision of the tumor that measured 33 cm, and we even performed an emasculation procedure due to invasion of the external genitals. This is the largest such tumor described in the literature [14–16]. Since wide excisions usually cause noteworthy distortion and leave patients with serious cosmetic problems, reconstructive surgery may be required in almost every instance to restore tissue defects using a local skin flap, skin graft, or myocutaneous flap.

In cases in which primary flap reconstruction is performed simultaneously with tumor excision, there should be no doubt as to the adequacy of the excision, since the presence of a flap may prevent the detection of local recurrence. In general, the reconstructive challenge in DFS such as in the trunk, extremities, head, and neck is related to large tissue defects which need covering when vital structures are exposed [17, 18]. In our case, a local anterolateral thigh skin flap was chosen in the pelvis to cover the defect caused by the emasculation, and on the abdominal wall, we chose a thin skin graft.

As for adjuvant treatment, imatinib mesylate can be used to treat unresectable, recurrent, and/or metastatic disease in patients with a t(17;22) translocation [19]. In cases of positive or inadequate margins, recurrence, or unacceptable functional or cosmetic results after wide excision, radiotherapy can be combined with surgery [19, 20]. A combination of conservative excision and adjuvant radiotherapy was reported to reduce the rate of local recurrence by 5% [12].

## Conclusion

DFS is a malignant fibroblastic tumor with a high potential for recurrence. The pelvic location is not common in the literature, and wide excision in this area requires reconstructive surgery to cover the defects.

Our case was extraordinary due to several aspects: we report the largest size of DFS in the pelvic area (33 cm × 17 cm × 7 cm), which itself represents a rare location for this tumor. Our patient underwent wide surgical excision for this tumor. Two types of reconstruction were used to cover the enormous defect.

## Data Availability

Data supporting our findings were taken from the patient’s records.
